# Dietary energy drives the dynamic response of bovine rumen viral communities

**DOI:** 10.1186/s40168-017-0374-3

**Published:** 2017-11-28

**Authors:** Christopher L. Anderson, Matthew B. Sullivan, Samodha C. Fernando

**Affiliations:** 1 School of Biological Sciences, University of Nebraska, Lincoln, NE 68588 USA; 2 Departments of Microbiology, and Civil, Environmental and Geodetic Engineering, The Ohio State University, Riffe Building 266, 496 W 12th Ave, Columbus, OH 43210 USA; 3 Department of Animal Science, University of Nebraska-Lincoln, C220K Animal Science Complex, Lincoln, NE 68583-0908 USA

**Keywords:** Rumen, Viral metagenome, Viral diversity, Phage ecology, Auxiliary metabolic genes

## Abstract

**Background:**

Rumen microbes play a greater role in host energy acquisition than that of gut-associated microbes in monogastric animals. Although genome-enabled advancements are providing access to the vast diversity of uncultivated microbes, our understanding of variables shaping rumen microbial communities is in its infancy. Viruses have been shown to impact microbial populations through a myriad of processes, including cell lysis and reprogramming of host metabolism. However, little is known about the processes shaping the distribution of rumen viruses or how viruses may modulate microbial-driven processes in the rumen. To this end, we investigated how rumen bacterial and viral community structure and function responded in five steers fed four randomized dietary treatments in a crossover design.

**Results:**

Total digestible nutrients (TDN), a measure of dietary energy, best explained the variation in bacterial and viral communities. Additional ecological drivers of viral communities included dietary zinc content and microbial functional diversity. Using partial least squares regression, we demonstrate significant associations between the abundances of 267 viral populations and variables driving the variation in rumen viral communities. While rumen viruses were dynamic, 14 near ubiquitous viral populations were identified, suggesting the presence of a core rumen virome largely comprised of novel viruses. Moreover, analysis of virally encoded auxiliary metabolic genes (AMGs) indicates rumen viruses have glycosidic hydrolases to potentially augment the breakdown of complex carbohydrates to increase energy production. Other AMGs identified have a role in redirecting carbon to the pentose phosphate pathway and one carbon pools by folate to boost viral replication.

**Conclusions:**

We demonstrate that rumen bacteria and viruses have differing responses and ecological drivers to dietary perturbation. Our results show that rumen viruses have implications for understanding the structuring of the previously identified core rumen microbiota and impacting microbial metabolism through a vast array of AMGs. AMGs in the rumen appear to have consequences for microbial metabolism that are largely in congruence with the current paradigm established in marine systems. This study provides a foundation for future hypotheses regarding the dynamics of viral-mediated processes in the rumen.

**Electronic supplementary material:**

The online version of this article (10.1186/s40168-017-0374-3) contains supplementary material, which is available to authorized users.

## Background

Over 3.6 billion domesticated ruminants graze an estimated 26% of our planet’s ice-free terrestrial land [1] while accounting for 14.5% of anthropogenic greenhouse gas emissions globally [2]. As the need for meat and milk increases with the growing population density [1], ruminant agriculture is expected to increase leading to a concurrent rise in greenhouse gas emissions. Central to grazing ruminants are the nutrient and energy transformations in the rumen that are driven by a complex community of largely uncharacterized microbes [3]. This microbial consortia can utilize a broad range of substrates including cellulose-rich substrates of mainly indigestible plant material and convert it into energy sources such as volatile fatty acids (VFAs) and microbial cell protein for host absorption and metabolism [4, 5]. The synergistic actions of rumen microbes and assimilation of fermentation end products account for ~ 70% of the ruminant animal’s energy needs [6]. This is far greater than the contribution of hindgut fermentation to energy acquisition in monogastric mammals (~ 10–30%) [6]. Due to the influence of rumen microbes on host nutrient acquisition, rumen microbial community composition has been linked to host feed efficiency [7] and animal productivity measures [8, 9]. While there is a growing understanding of the importance of rumen microbes in regard to ruminant health and productivity, the roles of viruses in shaping rumen microbial communities and in turn ecosystem function are not well established.

Viruses influence microbial community composition and function through a number of processes, including both top-down and bottom-up effects resulting from cell lysis, horizontal gene transfer, metabolic reprogramming, and active lysogeny [10–15]. Multitrophic ecosystem modeling of surface oceans has demonstrated viral-mediated lysis increases recycling of organic matter and net primary productivity [16]. In addition to the effects of predation, viruses often acquire host-derived auxiliary metabolic genes (AMGs) that augment and redirect cell metabolism to bolster viral replication [17, 18]. A recent study using a metabolomic approach demonstrated that alterations in *Pseudomonas aeruginosa* metabolism were specific to the infecting virus [19]. The distinct metabolic responses observed were linked to AMGs and in general were attributed to the vast viral genetic diversity that arises from recombination and exchange of functional modules [19, 20]. Considering that viruses infect a large fraction of microbes at any given time [21], metabolic reprogramming by viruses has the potential to modify microbial processes on an ecosystem scale.

Whether it is a direct or indirect effect, there is growing evidence suggesting environmental conditions have a large influence on viral population dynamics and, as a result, on the viral-driven processes outlined above [22–30]. Viral communities in marine systems have been shown to significantly vary with depth, latitude, temperature, season, oxygen content, and microbial density [22, 23, 29]. Gut viral metagenomes investigated in humans and mice (hindgut fermenters) have been characterized as possessing large inter-individual variation that remains fairly stable over time, yet viral communities from these hosts are also sensitive to short- and long-term dietary perturbations [24–27]. In ruminants, studies investigating the rumen virome are limited. A single study in sheep using pulse-field gel electrophoresis has suggested a potential influence of diet on rumen viruses [31]. However, metagenomics would provide a deeper understanding of the potential effect of diet on rumen viruses. Those that have utilized a metagenomic approach to study rumen viruses have focused on characterizing the composition and variation in the virome between animals fed the same diet and not identified ecological drivers of viral communities [32–34]. These previously published metagenomes indicate that rumen viruses potentially encode AMGs associated with a variety of pathways. However, it is not clear if the viral metabolic signatures in these datasets are the result of cellular contamination. Thus, with the growing understanding of how viruses impact nutrient fluxes and microbial metabolism, and the critical roles of rumen microbes in host health and productivity, it is imperative to better characterize the processes shaping microbial-viral interactions and the roles of AMGs in metabolic reprogramming in the rumen ecosystem. Here, we utilized five steers fed four different diets in a crossover design to investigate how rumen microbial and viral communities simultaneously respond to dietary perturbation via 16S rRNA gene amplicon and metagenome sequencing. This approach helped identify ecological drivers of viral and bacterial populations and uncover AMGs from bona fide viral contigs to detail how abundant viruses may manipulate biochemical process in the rumen.

## Results and discussion

To assess the impact of diet on bovine rumen microbial and viral communities, we fed five steers four different diets in a 5 × 4 row-column crossover design (Table 1). Diets differed in energy content, nutritional composition, and fiber components (Table 2). Rumen pH differed by diet (*P* = 0.002, *F* = 11.540, three-way ANOVA considering effects of diet, period, and host animal; Additional file 1), while total VFAs altered by host animal (*P* = 0.016, *F* = 5.496) and period (*P* = 0.021, *F* = 5.431), but not diet (*P* = 0.110, *F* = 2.686). Bacterial 16S rRNA gene amplicons and microbial metagenomes from the 20 rumen samples alongside 15 viral metagenomes were sequenced to quantify changes in the microbial and viral communities in response to dietary change (Additional file 2). Viral metagenomes were constructed from unamplified DNA to reduce amplification bias. The viral metagenomes were only available for a subset of the microbial dataset, periods 2–4 of the study (Table 1), but were highly pure as the maximum cellular contamination in any viral metagenome was 0.110% (mean 0.043%; Additional file 2), below the 0.2% threshold currently accepted in the literature [35]. Additionally, six viral enrichments from the two most contrasting diets with respect to dietary energy (55CS and 27CDS diets; Table 2) were subjected to deeper sequencing (1.39 Gbp/sample; Additional file 2). To differentiate these metagenomes from the previously described viral metagenomes, we refer to this additional dataset as the deep viral metagenomes. Maximum cellular contamination in the deep viral metagenomes was 0.249% (mean 0.108%; Additional file 2).

**Table 1:** Experimental design and rumen sampling for microbial and viral community analyses

Steer identifier	Period 1	Period 2	Period 3	Period 4
346	27CDS^M^	Corn^M,V^	40MDGS^M,V^	55CS^M,V,DV^
3244	27CDS^M^	Corn^M,V^	40MDGS^M,V^	55CS^M,V,DV^
222	55CS^M^	40MDGS^M,V^	Corn^M,V^	27CDS^M,V,DV^
3257	40MDGS^M^	27CDS^M,V,DV^	55CS^M,V,DV^	Corn^M,V^
259	Corn^M^	55CS^M,V^	27CDS^M,V,DV^	40MDGS^M,V^

Five steers were rotated through four diets in a row-column design. Microbial metagenomes and 16S rRNA amplicons (M) were sequenced from all periods, viral metagenomes from periods 2–4 (V), and deep viral metagenomes were sequenced from select 27CDS and 55CS samples (DV)

**Table 2:** Composition of dietary treatments

	Corn	27CDS	40MDGS	55CS
High-moisture corn	51.25	36.30	28.50	–
Dry-rolled corn	36.25	24.20	19.00	–
Condensed distillers solubles	–	27.00	–	–
Modified distillers grains plus solubles	–	–	40.00	40.00
Silage	–	–	–	55.00
Brome	7.50	7.50	7.50	–
Supplement	5.00	5.00	5.00	5.00
Relative feed value	1100.14	1378.78	712.65	252.00
Total digestible nutrients	81.43	82.65	78.50	73.30
Acid detergent fiber	5.39	4.91	9.28	15.25
Neutral detergent fiber	11.22	10.14	19.25	25.45
Crude protein	8.74	12.16	17.96	17.17
Nitrate (ppm on dry mater basis)	25.75	25.75	25.75	19.00
Ca	0.08	0.07	0.09	0.18
P	0.34	0.82	0.60	0.56
K	0.63	1.33	0.97	1.25
Mg	0.14	0.31	0.23	0.25
Zn	24.90	123.47	38.51	40.15
Fe	55.91	92.93	125.22	197.25
Mn	10.84	18.58	16.32	21.60
Cu	2.57	4.16	4.14	6.52
S	0.11	0.23	0.26	0.26
Na	0.03	0.17	0.17	0.13
Mo	0.24	0.38	0.51	0.51

Ingredient inclusions and chemical composition for dietary treatments (expressed as percent dry matter)

### Overview of rumen viral and bacterial community composition

**Fig. 1:**
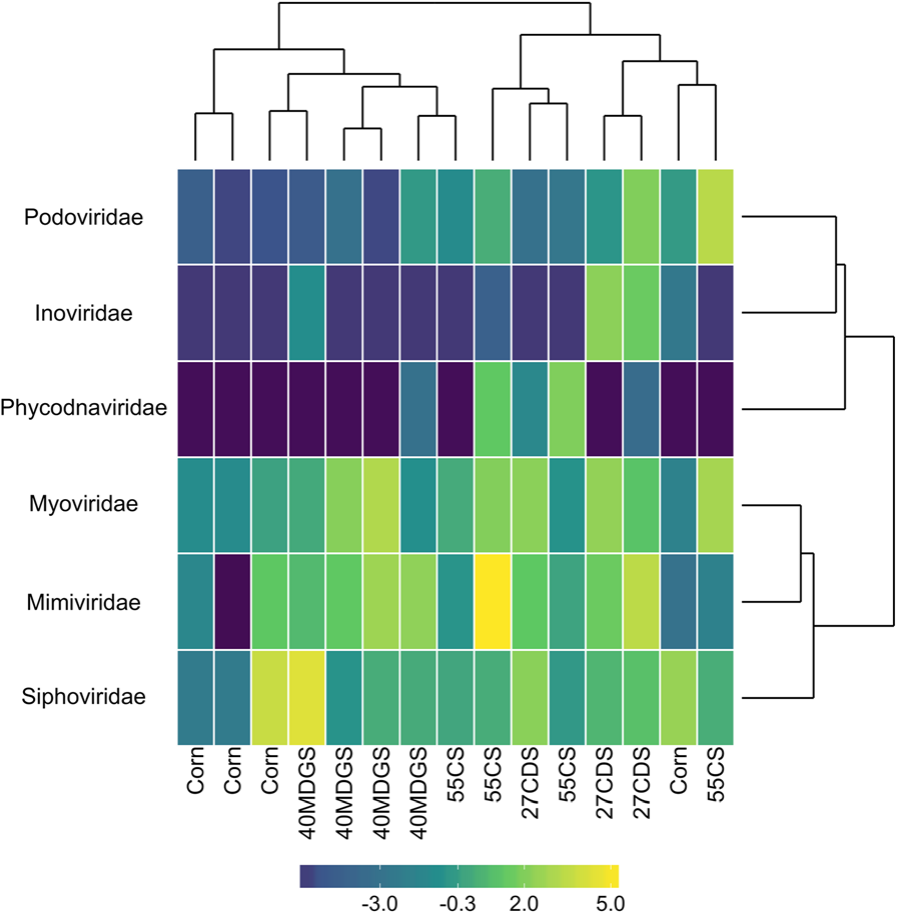
Normalized abundances (log_2_ counts per kilobase per million reads) of viral families identified in rumen viral metagenomes. Genome-binning of contigs resulted in 2243 viral populations (see the “Methods” section), of which 118 displayed homology to viruses in the RefSeq database. Viral populations with homology to known viruses accounted for 2.4% of all viral metagenome reads. Viral annotations were assigned based on nucleotide similarity of contigs to the viral RefSeq database (BLASTN, E-value threshold of 10^−5^, bit-score threshold of 50). 27CDS—27% condensed distillers solubles; 40MDGS—40% modified distillers grains plus solubles; 55CS—55% corn silage

**Fig. 2:**
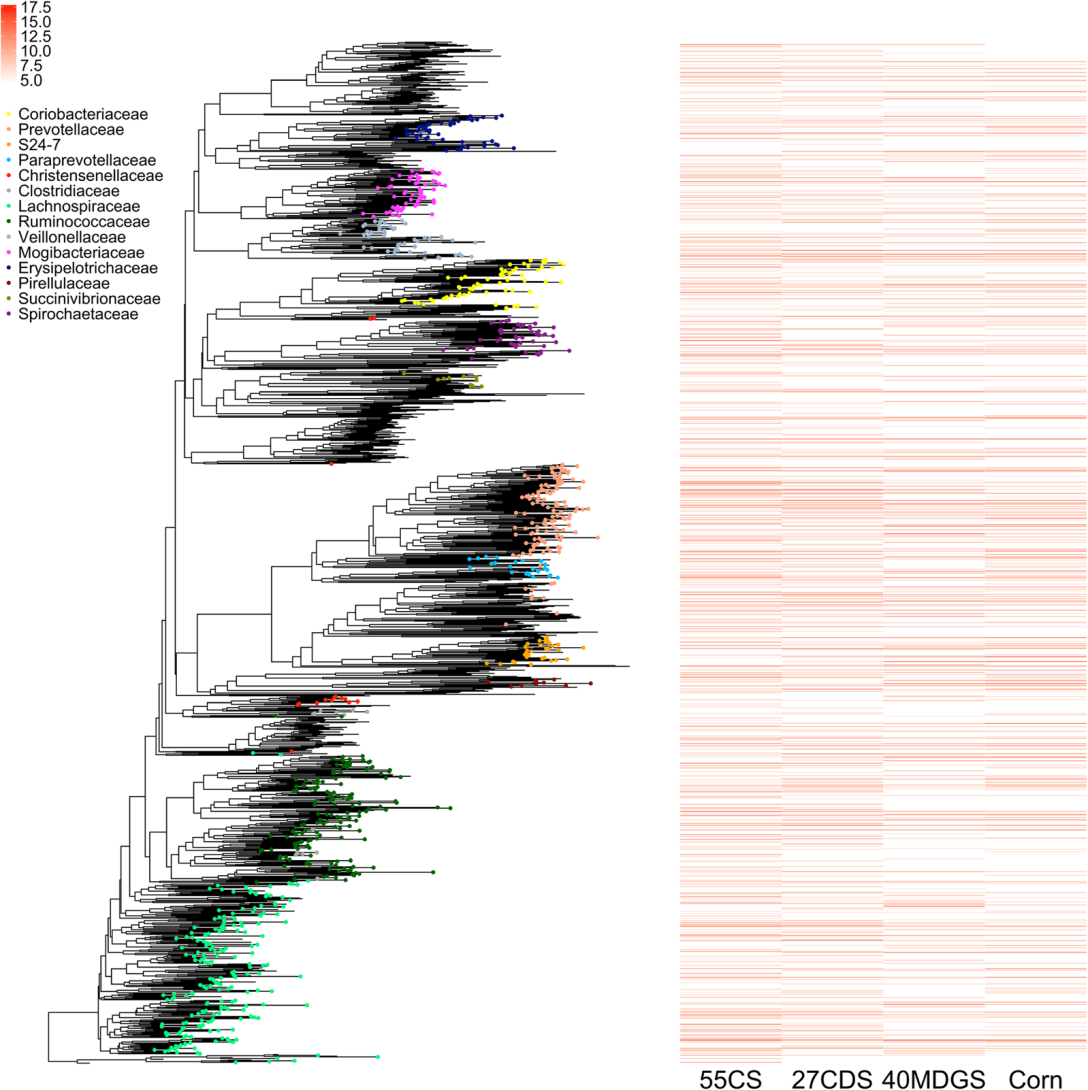
Phylogenetic relationships and normalized median diet abundances (log_2_ counts per million reads) of identified OTUs. Families with a maximum abundance greater than 3% in a sample are denoted on the phylogenetic tree. The most abundant bacterial families included *Rumminococcaceae*, *Lachnospiraceae*, and *Prevotellaceae*, in agreement with previous studies of the rumen microbiome [3]

Similar to recent analyses of viral communities [30], contigs generated from a cross-assembly of reads from the viral metagenomes and a cross-assembly of the deep viral metagenomes were filtered using VirFinder [36] and VirSorter [37]. The resulting viral contigs were binned to delineate viral populations in a manner that maximized the number of bins with a single copy of the *terL* gene and minimized potential microbial contamination (see the “Methods” section for full details on binning and filtering procedures). This genome-binning process resulted in the identification of 2243 viral populations with a cumulative bin length greater than 10 kbp. A median 34% of sample reads were recruited to viral populations. No differences were observed between diets in the number of reads recruited to populations (*P* = 0.629, ANOVA). The contigs of viral populations were assessed for homology to viral RefSeq genomes. Of the 2243 viral populations, 118 (5.3%) had significant similarity to a known virus. Based on taxonomic affiliations, 108 viral populations were identified as having a eubacterial host and 10 with a eukaryotic host. None of the viral populations were characterized as archaeal viruses. This may be due to the low abundance of *Archaea* in the rumen [38] and the poor representation of archaeal viruses in databases [39]. Family-level annotations were summarized from the taxonomic assignments of viral populations. Abundant viral families included *Myoviridae*, *Siphoviridae*, *Mimiviridae*, and *Podoviridae* (Fig. 1), in agreement with previous investigations of the rumen virome [32]. While *Mimiviridae* viruses have been observed in rumen viral metagenomes [32], due to their large sizes we would have expected these viruses to be excluded from viral enrichments generated through tangential flow filtration with a 0.2 μm filter [40]. Contigs annotated as *Mimiviridae* may originate from smaller novel viruses that infect rumen protozoa and have genomic regions with homology to *Mimiviridae* viruses. For example, virophages of *Mimiviridae* have genes that show high similarity to members of the host family [41]. To monitor concurrent changes in bacterial community dynamics, the V4 hypervariable region of the 16S rRNA gene was sequenced. A total of 1311 OTUs were identified across the 20 rumen samples, with a minimum depth of 21,228 reads per sample. Taxonomy could be confidently assigned to ~ 80% of the OTUs at family level (Fig. 2) and only ~ 50% at the level of genera. Families in high abundance included *Ruminococcaceae* and *Lachnospiraceae* of the phlyum *Firmicutes* and *Prevotellaceae* of the phylum *Bacteroidetes*. These phyla are commonly associated with the rumen ecosystem [3].

### Diet and host animal influence the population and functional response of rumen microbial and viral communities

**Fig. 3:**
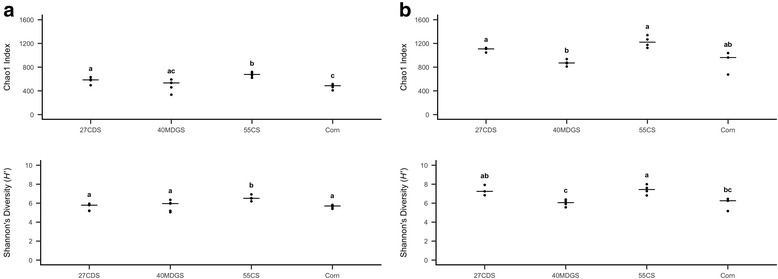
Alpha diversity comparisons of rumen bacterial OTUs and viral populations across diets. Species richness (Chao1 index) and diversity (Shannon’s diversity index) were found to significantly differ by diet in bacterial (**a**) and viral (**b**) communities (*P* < 0.05, three-way ANOVA considering the effects of diet, period, and steer). The combinations of diets that differed in richness and diversity in the bacterial and viral datasets were unique (*P* < 0.05, post-hoc pairwise *t* tests). No differences were observed in bacterial or viral alpha diversity metrics based on host animal or period. Letters denote differences in richness and diversity observed when comparing the alpha diversity metrics using pairwise comparisons of diets. 27CDS—27% condensed distillers solubles; 40MDGS—40% modified distillers grains plus solubles; 55CS—55% corn silage

Using the abundances of viral populations and bacterial OTUs across samples, we explored the global effects of diet on viral and bacterial diversity in the rumen. Rarefaction curves of species richness (Chao1 index) displayed a plateauing effect with increasing sequencing depth, suggesting adequate sequencing depth to investigate the dominant viral and bacterial populations (Additional file 3). When abundances were subsampled to an even depth, a significant shift in OTU and viral population richness (Chao1 index) and diversity (Shannon’s diversity index) was observed across diets (*P* < 0.05, three-way ANOVA considering the effects of diet, period, and steer; Fig. 3). Further, distinct sets of diets were found to differ in species richness and diversity for the bacterial and viral populations (*P* < 0.05, post-hoc pairwise *t* tests; Fig. 3). No differences in richness or diversity were observed in the bacterial or viral communities based on steer or period (*P* > 0.05, three-way ANOVA).

**Fig. 4:**
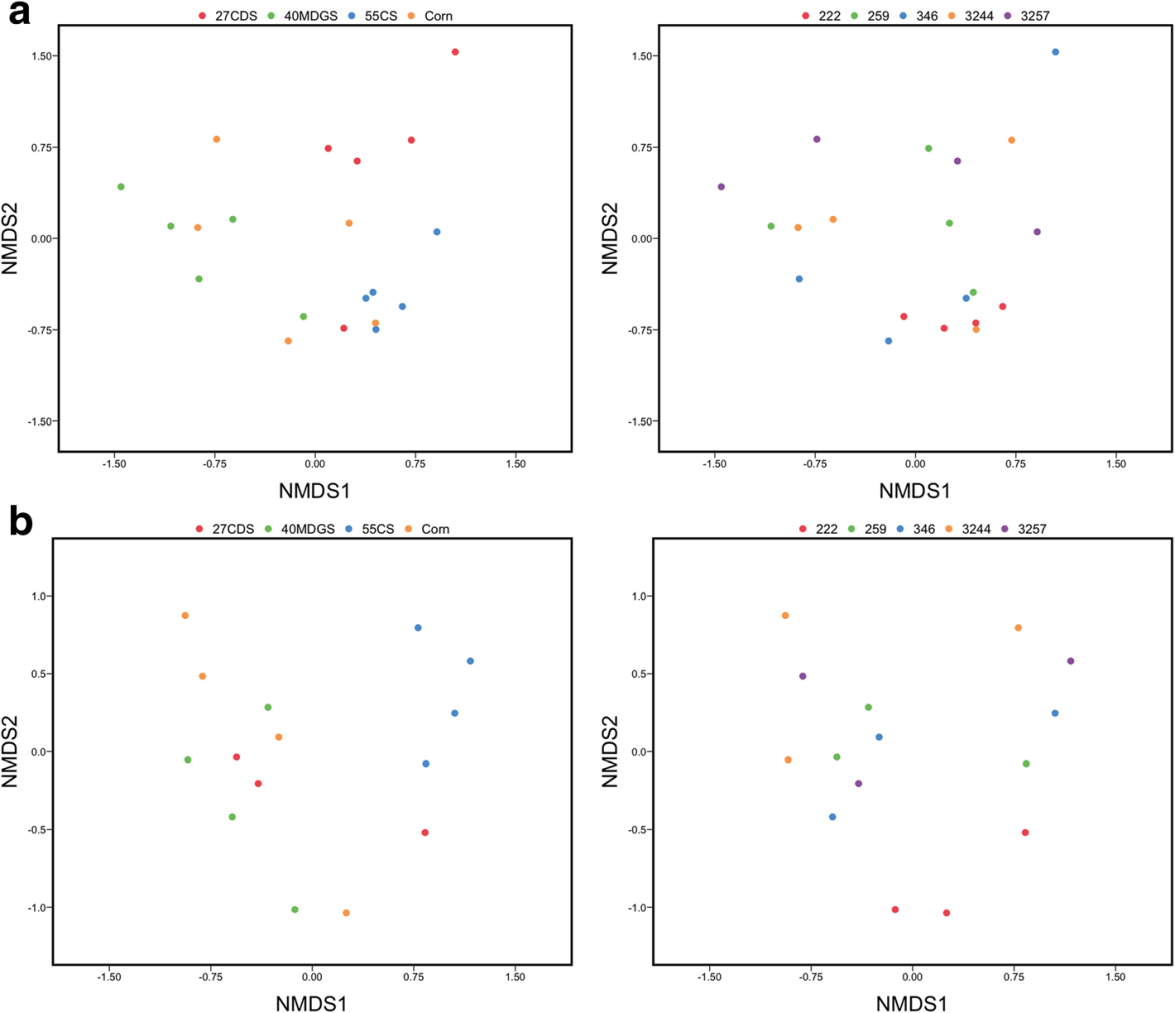
Unconstrained NMDS ordination analysis was used to visualize beta diversity based on OTU (**a**) and viral population (**b**) abundances across samples. PERMANOVA results indicate that structuring of bacterial communities is best explained by diet (diet: *P* = 0.001, *R* ^2^ = 0.365; period: *P* = 0.188, *R* ^2^ = 0.140; steer: *P* = 0.312, *R* ^2^ = 0.166). Both dietary and host animal effects were found to significantly explain the variation in viral populations (diet: *P* = 0.002, *R* ^2^ = 0.266; period: *P* = 0.096, *R* ^2^ = 0.145; steer: *P* = 0.048, *R* ^2^ = 0.290). 27CDS—27% condensed distillers solubles; 40MDGS—40% modified distillers grains plus solubles; 55CS—55% corn silage. 222, 259, 346, 3244, and 3257 represent animal identifiers

**Fig. 5:**
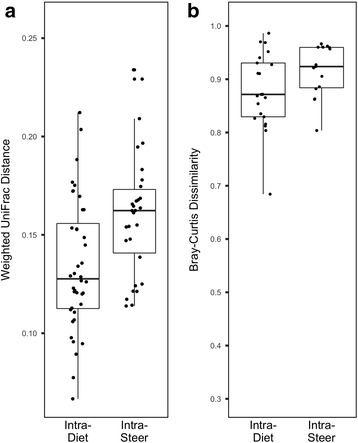
Analysis of intra-diet and intra-steer distances and dissimilarities reveals a larger influence of host-associated factors on viral populations compared to bacterial OTUs. **a**) Within diet weighted UniFrac distances were significantly lower than within animal weighted UniFrac distances in the rumen bacterial communities (*P* = 0.001, *t* = − 3.366, *t* test). **b**) No statistically significant differences were observed between intra-diet and intra-steer Bray-Curtis dissimilarities in viral populations (*P* = 0.067, *t* = − 1.896, *t* test)

To address dietary and host effects on beta diversity, weighted UniFrac distance and Bray-Curtis dissimilarity matrices were generated from normalized abundance of bacterial OTUs and viral populations, respectively. A PERMANOVA analysis [42, 43] considering the effects of diet, period, and host animal indicated structuring of rumen bacterial communities to be largely controlled by dietary factors (diet: *P* = 0.001, *R* ^2^ = 0.365; period: *P* = 0.188, *R* ^2^ = 0.140; steer: *P* = 0.312, *R* ^2^ = 0.166; Fig. 4a). The observed variation within the viral populations, on the other hand, was significantly associated with diet and host animal in which the sample was collected from (diet: *P* = 0.002, *R* ^2^ = 0.266; period: *P* = 0.096, *R* ^2^ = 0.145; steer: *P* = 0.048, *R* ^2^ = 0.290; Fig. 4b). To support these findings, we compared intra-steer dissimilarities and distances with intra-diet dissimilarities and distances (Fig. 5). This analysis revealed intra-steer distances to be significantly higher than intra-diet distances in the bacterial communities (*P* = 0.001, *t* = − 3.366, *t* test; Fig. 5a). No such difference was observed in the viral populations (*P* = 0.067, *t* = − 1.896, *t* test; Fig. 5b).

To build upon our results based on OTUs and viral populations, we next clustered open reading frames (ORFs) from the microbial and viral metagenomes into protein clusters (PCs) and investigated the functional response of the viral and bacterial communities to dietary change. Viral PCs were again defined from contigs identified as viral by a combination of VirFinder [36] and VirSorter [37]. After removing rare and low abundant PCs, 321,513 microbial PCs and 81,359 viral PCs were identified for downstream analyses. Many rare rumen microbial and viral PCs were underrepresented, and deeper sequencing efforts in the future may help investigate the role of such PCs on bacterial and viral ecology in the rumen. The diversity of PCs (Shannon diversity index) did not differ in microbial datasets, but a significant shift associated with diet was observed in viral PC diversity (three-way ANOVA considering the effects of diet, period, and steer; Additional file 4). To address dietary and host effects on functional beta diversity, Bray-Curtis dissimilarity matrices were calculated from the relative abundances of PCs. A PERMANOVA analysis [42, 43] considering the effects of diet, period, and host animal indicated structuring of rumen microbial communities to be largely driven by dietary factors, with host animal in which the sample was collected from explaining a non-significant portion of the observed variation (diet: *P* = 0.001, *R* ^2^ = 0.259; period: *P* = 0.222, *R* ^2^ = 0.146; steer: *P* = 0.081, *R* ^2^ = 0.222; Additional file 5). Both dietary and host effects were identified as significant factors influencing viral PCs (diet: *P* = 0.001, *R* ^2^ = 0.270; period: *P* = 0.083, *R* ^2^ = 0.143; steer: *P* = 0.047, *R* ^2^ = 0.288; Additional file 5). Beta diversity comparisons based on PCs are largely in agreement with those obtained using viral populations and bacterial OTUs.

Bacterial composition across ruminant species has been shown to vary based on dietary and host factors, with diet being the most influential factor [3]. Previously, diet was found to influence banding patterns generated through pulse-field gel electrophoresis on a viral enrichment [31]. In comparison, the metagenomic approach used in the current study provides improved resolution into the response of viruses to dietary change. Others that have taken a metagenomic approach to study rumen viral communities have explored the variation between animals on the same diet [32–34]. Consequently, prior to the current study, the relative importance of dietary factors in structuring rumen viral communities had not been demonstrated. Diet has been identified as a factor that drives fecal viral communities in mice and humans [25, 27]. In mice, divergent responses of microbes and viruses to dietary change have been described [27]. Patterns we observed in rumen viral alpha and beta diversity are in agreement with this finding. The diet combinations found to vary in species richness and diversity within the bacterial and viral communities were different. Additionally, there appears to be a larger influence of host animal effects on the distribution of viral populations and PCs than was observed for the bacterial OTUs and microbial PCs. Large inter-individual variation has been characterized in host associated viromes, including the rumen [24, 25, 33]. These findings are potentially suggestive of a direct dietary or host-related effect on rumen viruses, independent of the microbial populations. The use of a washout diet [27] may provide a powerful approach to further explore the resilience of rumen viruses to better elicit the mechanisms underlying the responses of viruses and microbes to dietary perturbation.

### Total digestible nutrients, zinc, and microbial functional diversity are ecological drivers of rumen viruses

**Fig. 6:**
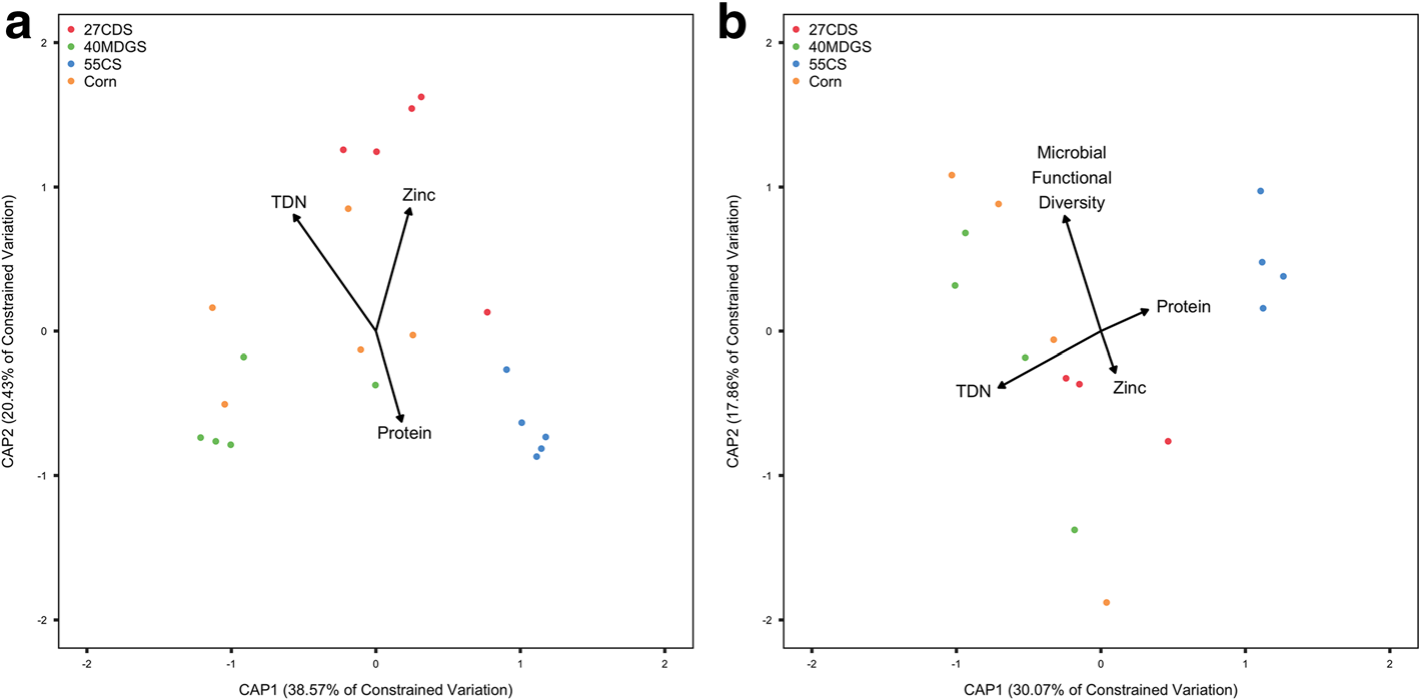
Ordination of CAP analysis displaying the factors influencing rumen bacterial and viral communities. The CAP model with TDN, protein, and zinc as independent variables explained a significant portion of the variation in bacterial OTUs (*P* = 0.001, *F* = 2.736, variation explained = 33.9%, ANOVA permutation test for CAP) (**a**). Subsequent testing of the marginal effects of each term found TDN (*P* = 0.001, *F* = 4.342), zinc (*P* = 0.002, *F* = 3.587), and protein (*P* = 0.012, *F* = 2.379) to vary significantly with the microbial communities. Backward selection identified TDN, protein, zinc, and microbial functional diversity (Shannon’s diversity index) as potential drivers of rumen viral populations (**b**). The CAP model including these independent variables explained a significant portion of the variation in viral metagenomes (*P* = 0.002, *F* = 1.383, variation explained = 35.6%, ANOVA permutation test for CAP). Investigation of the marginal effects of each variable revealed TDN (*P* = 0.001, *F* = 1.936), zinc (*P* = 0.025, *F* = 1.482), and microbial functional diversity (*P* = 0.022, *F* = 1.402) to be the predominant factors influencing rumen viral populations. 27CDS—27% condensed distillers solubles; 40MDGS—40% modified distillers grains plus solubles; 55CS—55% corn silage

After identifying diet as a driver of bacterial and viral communities, we examined which dietary components and parameters explain a significant portion of the variation observed in bacterial OTUs and viral populations. To account for potential effects of multicollinearity between explanatory variables, we filtered factors by imposing a Pearson correlation coefficient threshold of 0.85 and selecting factors of ecological interest. This resulted in an initial model including total digestible nutrients (TDN), zinc, protein, pH, VFA concentrations, species diversity and richness, and functional diversity. Species richness and diversity were based on abundances of OTUs and viral populations, while functional diversity metrics were based on PCs. Bacterial diversity metrics were used as potential explanatory variables for viral communities and vice versa for bacterial communities. Backward stepwise selection of variables was then performed in terms of a constrained analysis of principal coordinates (CAP) model. Bray-Curtis dissimilarities calculated from normalized OTU and viral population abundances were used in the CAP model. During backward selection, variables with *P* > 0.10 were removed from the model. Backward selection identified TDN, zinc, and protein as potential explanatory variables for rumen bacterial communities. In comparison, TDN, zinc, protein, and microbial functional diversity (Shannon diversity index) were identified as potential explanatory variables of viral communities. Factors in the two models above had a variance inflation factor less than 5, indicating minimal multicollinearity among the explanatory variables. The CAP models using variables identified by backward selection explained a significant portion of the variation in bacterial OTUs (*P* = 0.001, *F* = 2.736, variation explained = 33.9%, ANOVA permutation test for CAP; Fig. 6a) and viral populations (*P* = 0.002, *F* = 1.383, variation explained = 35.6%, ANOVA permutation test for CAP; Fig. 6b). We further assessed the marginal effects of each term in the CAP models. TDN (*P* = 0.001, *F* = 4.342), zinc (*P* = 0.002, *F* = 3.587), and protein (*P* = 0.012, *F* = 2.379) were significant explainers of bacterial community variation. In viral populations, TDN (*P* = 0.001, *F* = 1.936), zinc (*P* = 0.025, *F* = 1.482), and microbial functional diversity (*P* = 0.022, *F* = 1.402), but not protein (*P* = 0.169, *F* = 1.170), were identified as significant ecological drivers.

Contrary to the wording of the term, TDN values are not a global indicator of nutrient content because the metric does not take into account all types of nutrients. Instead, TDN content is an energy index based on measures of the digestible fiber, protein, lipid, and carbohydrate components in a diet [44]. We cannot eliminate collinear effects between explanatory variables, and therefore, a covariate may be the source of causal influence. Accordingly, ecological drivers should be considered in terms of their covariates. For instance, TDN had a strong negative relationship with acid detergent fiber (*r* = − 0.99) and neutral detergent fiber (*r* = − 0.98) content across diets. TDN was also correlated with the measured calcium (*r* = − 0.96) and iron (*r* = − 0.93) in the diets, but these factors are likely to be less ecologically important than the changes in rumen physiology caused by altered fiber and energy content.

Following identification of ecological drivers of rumen bacterial and viral populations, we used partial least squares regression (PLSR) [45] to model linear associations between explanatory variables and normalized OTUs and viral population abundances. PLSR identified 172 bacterial OTUs and 267 viral populations which explained a large proportion of the response (greater than 30% variation explained) in the first component of the PLSR model. Univariate linear regression analysis was used to support the strength and direction of associations identified in the PLSR model. Linear regressions demonstrated 156 of the 172 bacterial OTUs and 264 of the 267 viral populations to have significant but noisy correlations with at least one of the explanatory variables (*P* < 0.05; Additional file 6). A majority of the significant bacterial OTUs and viral populations had a negative correlation with TDN, while fewer correlations were identified with protein, zinc, and microbial diversity.

Dietary fiber content (and thus TDN, due to the large negative correlation between fiber content and TDN) has a large influence on rumen bacterial and eukaryotic microbial populations [5, 46–49]. In addition to TDN, zinc and microbial functional diversity were identified as important ecological drivers of rumen viruses (Fig. 6). Zinc is integral to many protein functions. Most metalloproteases contain zinc [50], and rumen bacteria produce an abundance of metalloproteases [51]. As such, zinc likely plays an important role in rumen bacterial enzyme function and in-turn rumen metabolism. This is emphasized by the change in bacterial OTUs with zinc dietary content. The roles of other divalent cations in shaping rumen microbial communities should be considered, but are difficult to explore in the current analysis due to their covariate nature. A change in microbial host abundance brought on by changes in zinc content may in turn influence the abundance of viruses associated with those hosts. This potential indirect effect of ecological drivers on viral community structure should be considered for all factors identified. Viruses require hosts, whose composition is controlled by dietary conditions, and thus, the ecological drivers of microbes may also impact viral communities. This seems plausible in the current study given bacterial and viral communities both varied significantly with dietary TDN and zinc content. On the contrary, some factors are known to directly impact viral function. For instance, viruses with a membrane are more influenced by salinity than non-lipid containing viruses [52]. Other unidentified factors altering viral function that are imposed by dietary conditions may be at play in the rumen. We noted that viral populations were significantly influenced by the diversity of microbial PCs, but not richness or diversity of bacterial OTUs. Since microbial PCs were not significantly different by diet (Additional file 4) and as a weak correlation was identified between microbial PC diversity and TDN (Pearson correlation = 0.75), we believe that the influence of PC diversity on viral populations may be an artifact of the relationship between PC diversity and TDN. Future work on rumen viral-host dynamics should consider the relationship between microbial density, viral counts, and viral lifestyle [53]. A shift in viral lifestyle driven by dietary factors could have profound impacts on rumen microbial communities, including a relative decrease in viral predation when microbial densities are high and selective mortality upon induction of prophages [28, 53, 54].

**Table 3:** Core viral populations

Viral population	Bin length (kbp)	Median coverage (%)	Taxonomy	Predicted host	Max sample reads recruited (%)	Mean sample reads recruited (%)
1052	15.8	87.3	–	–	0.82	0.15
1566	11.9	73.2	–	–	0.33	0.07
1731	11.0	90.2	–	*Bacteroidetes*	0.66	0.16
614	26.8	34.2	–	–	0.32	0.07
769	21.2	58.3	–	–	0.25	0.06
1507	12.4	43.7	–	–	0.22	0.03
717	22.6	74.6	–	–	1.30	0.22
123	104.4	94.2	–	*Proteobacteria*	6.59	1.24
477	33.9	87.7	–	*Proteobacteria*	0.61	0.21
726	22.2	70.1	–	–	2.49	0.32
910	17.9	72.8	–	–	0.40	0.08
1127	14.9	45.3	–	–	0.26	0.05
1128	14.9	66.1	–	–	0.21	0.04
1822	10.5	66.2	–	–	0.29	0.05

Core viral populations were defined as having at least 15% coverage in 80% or more of the rumen viral metagenomes. Since none of the core populations had homology to known viruses, hosts were inferred using HostPhinder [55]

### Near ubiquitous viral populations suggest the presence of a core rumen virome

Fourteen viral populations with at least 15% coverage in a sample were found in a minimum of 80% of rumen viral metagenomes (Table 3). Individually, these potential core viral populations recruited a maximum 6% of a sample’s reads, while collectively encompassing up to 10% of the reads in a given sample. We attempted to predict the hosts of these viral populations since they had no homology to known viruses; however, this analysis was only able to infer hosts for three viral populations to *Bacteroidetes* and *Proteobacteria* [55]. Improved sequencing depth and a greater number of samples from both the microbial and viral fractions may allow for improved viral-host predictions via in silico approaches. Previously, in an assessment of the viral content of the rumen in 13 dairy cattle, five assembled contigs longer than 30 kbp were found across all animals. The presence of core viral populations needs to be confirmed in a larger cohort of animals and ideally across ruminant species. Due to the low sequencing depth of some samples in the current study, we postulate that the number of core viral populations may increase upon the addition of deeper sequenced rumen viral metagenomes.

While these core populations should be considered preliminary, the implications of a core rumen virome are potentially far reaching. It is tempting to speculate on the relationship between the core viral populations and previously identified core rumen microbiome, mainly encompassed by poorly characterized microbes belonging to *Prevotella*, *Butyrivibrio*, *Ruminococcus*, *Lachnospiraceae*, *Ruminococcaceae*, *Bacteroidales*, and *Clostridiales*. [3]. It has been posit that the core microbiota in ruminants avoid washout because these microbes are associated with high energy-yielding reactions during the breakdown of feedstuffs [3], faster doubling time, and may be necessary for efficient fermentation [56]. Thus, core viruses may play an important role in maintaining the structure, persistence, or function of the core microbiome and influence rumen fermentation. A similar hypothesis was put forward regarding core viral groups found in healthy human subjects whose abundances are decreased in individuals with irritable bowel disease, ulcerative colitis, and Crohn’s disease [57].

### Viral AMGs are predominantly involved in carbon, folate, queuosine, and sulfur metabolism

**Fig. 7:**
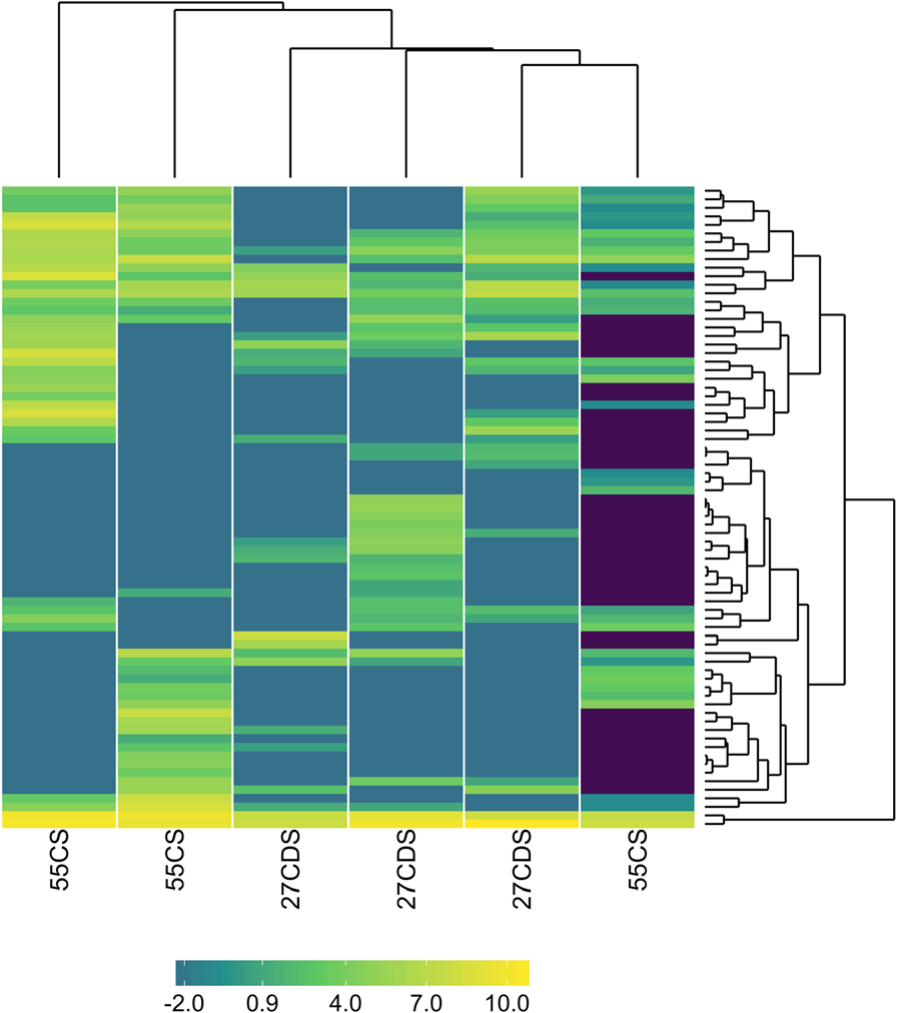
Normalized abundances (log_2_ counts per million reads) of virally encoded AMGs in deep viral metagenomes. The identification of AMGs was restricted to deep viral metagenome contigs from low (55CS) and high (27CDS) TDN diets that were longer than 1.5 kbp and identified as viral by VirSorter [61]. 27CDS—27% condensed distillers solubles; 40MDGS—40% modified distillers grains plus solubles; 55CS—55% corn silage

Based on results suggesting TDN to be the main ecological driver of the distribution of rumen viral populations, we sought out to examine the influence of TDN on viral AMG abundance. To this end, we mined the deep viral metagenome contigs assembled from 55CS (low TDN, high fiber content) and 27CDS (high TDN, low fiber content) diets for putative AMGs. We used VirSorter [37] to identify bona fide viral sequences from contigs longer than 1.5 kbp. The VirSorter pipeline as a virome decontaminator has precision greater than 98.99% [37], ensuring predicted AMGs are unlikely to be the result of cellular contamination. We restricted our analysis to the 75 AMGs (Additional file 7) present in Kyoto Encyclopedia of Genes and Genomes (KEGG) metabolic pathways and not normally associated with viral function (i.e., Class I AMGs [23], see the “Methods” section for details; Fig. 7). None of the AMGs were differentially abundant between the 55CS and 27CDS diets, and 21 (28.0%) were present in only one sample. If AMG composition is not controlled by diet, other selection factors may be at play in the acquisition and maintenance of AMGs in the rumen. Improved sequencing depth across a greater number of rumen viral enrichments is needed to elicit the factors at play in structuring AMG content. AMGs identified still highlight the roles for viruses in nutrient acquisition and metabolic reprogramming of microbial hosts in the rumen ecosystem. Below, we focus on detailing the potential roles of selected AMGs in rumen carbon, folate, queuosine, and sulfur metabolism.

Nearly all viral AMGs to date have been identified from marine environments, and while the rumen and ocean are disparate ecosystems with unique ecological drivers, marine AMGs can serve as a framework to understanding preliminary AMGs identified from the rumen and other gut environments. The current paradigm from marine studies is that carbon AMGs shift host microbial metabolism to mimic a state of starvation (for a review see [18]). In this model, host glycolysis is disrupted by the virally encoded glycogen synthase (*glgA*) resulting in a redistribution of carbon toward ribulose-5-phosphate (Ru5P) from glyceraldehyde-3-phosphate (GAP) and fructose-6-phosphate (F6P) for increased reducing power and deoxyribonucleotide (dNTP) biosynthesis during infection [14, 17]. Biosynthesis of dNTP is a limiting step in viral replication [58]. Other abundant marine AMGs in carbon metabolism are generally involved in harvesting and producing energy to fuel viral replication. It is proposed that these genes may overcome metabolic bottlenecks or be found in pathways that respond rapidly to environmental change in ways that direct metabolism away from amino acid biosynthesis and towards pathways favoring phage replication [17, 18].

Our results from the rumen largely agree with this marine-driven paradigm, but diverge in certain aspects. First, we observe *glgA* on viral contigs, implying rumen viruses redirect metabolic processes toward a similar physiological state seen during glucose deprivation [59]. In addition, the presence of fructose-1,6-bisphosphatase (*fbp*) and triosephosphate isomerase (*tpi*) in viruses is consistent with the change in carbon flux to F6P and GAP that can serve as carbon skeletons for nucleotide synthesis. We did not observe AMGs acting on F6P and GAP, as is seen in marine viromes [14, 17], but deeper sequenced rumen viral metagenomes may reveal these genes. Compared to marine viromes, we observed extensive reprogramming of carbon metabolism toward folate biosynthesis by rumen viruses. Tetrahydrofolate (THF) can serve as a one-carbon unit donor for diverse biological functions in three different states: 5-methyl-THF (important for the reaction of homocysteine to methionine), 5,10-methylene-THF (needed for the conversion of deoxyuridylate (dUMP) to deoxythymidine monophosphate (dTMP)), and 10-formyl-THF (a formyl donor in de novo purine biosynthesis reactions) [60, 61]. The first enzyme in the pterin branch of folate biosynthesis, GTP cyclohydrolase I (*folE*), along with other folate metabolism genes were found on viral contigs. These included dihydrofolate synthase (*folC*) and reductase (*folA*) which catalyze the conversion of dihydropteroate to dihydrofolate (DHF) and subsequent reduction to THF, respectively. Additionally, we detected methenyltetrahydrofolate cyclohydrolase (*folD*), which is responsible for the interconversion of 5,10-methylene-THF and 10-formyl-THF. Lastly, we found viral genes for glycine hydroxymethyltransferase (*glyA*), which encode the reversible interconversion of serine to glycine and THF to 5,10-methylene-THF. The diversion of carbon into one-carbon pools by folate has similar effects on host cell metabolism as shunting carbon through the pentose phosphate pathway (PPP)—both are important for nucleotide synthesis and generating reducing power. Folate metabolism genes have been observed in marine and human gut viral metagenomes before [24, 62], but not to the extent of the reprogramming of folate metabolism observed in this study from the rumen.

Another potential avenue in which rumen viruses might be aiding in the production of intermediates for viral replication is through fatty acid synthesis. Acetyl-CoA carboxylase (biotin carboxylase subunit, *accC*), encoding the rate-limiting step in fatty acid synthesis, along with 3-oxoacyl-(acyl-carrier-protein) synthase II (*fabF*) and III (*fabH*) were detected on viral contigs. Shifts towards fatty acid synthesis during viral infection are thought to be important for the recruitment of fatty acids to viral membrane assembly [63–65].

Carbon AMGs on rumen viral contigs relevant to energy production were phosphoglucomutase (*pgm*), phosphofructokinase (*pfkA*), and 5 glycosidic hydrolases (beta-glucosidase (*bglX*), alpha-glucosidase (*malZ*), alpha-amylase (*amyA*), maltooligosyltrehalose trehalohydrolase (*treZ*), and endoglucanase). The committed step of glycolysis, *pfkA*, should significantly aid in energy production during nutrient-limited conditions in the anaerobic rumen. We are unaware of any study that has reported *treZ* on a virus, but the remaining glycosidic hydrolases have been identified in viral genomes and metagenomes [66–69]. Glycosidic hydrolases catalyze the initial breakdown of complex carbohydrates, making them of particular importance for energy production in the rumen. Again though, we emphasize that despite the 27CDS diet being high in starch and 55CS in cellulose, we did not observe differences in the abundance of glycosidic hydrolases specific for starch and cellulose substrates. We hypothesize that these enzymatic steps represent metabolic bottlenecks to energy production specific to the rumen, and viral encoded proteins overcome these limitations to boost host metabolism during viral infection.

Abundant AMGs were also detected in queuosine and sulfur metabolism pathways. We identified all five enzymes involved in the conversion of GTP to preQ1, a precursor for queuosine nucleoside synthesis. Queuosine is a hypermodified guanosine found in the wobble position of tyrosine, histidine, aspartate, and asparagine tRNAs. Queuosine genes have been identified in both freshwater and marine ecosystems as well as *Streptococcus pneumoniae* [70–72]*.* It remains unknown why viruses carry queuosine metabolism genes, but it has been hypothesized that these genes act to increase translation efficiency of host protein synthesis or even their own protein synthesis as a feedback mechanism to control the production of phage structural proteins [71, 72]. The AMGs on the most contigs were cysteine desulfurase (*iscS*) and phosphoadenosine phosphosulfate reductase (*cysH*). These genes were found in all deep viral metagenomes in this study. Proteins involved in *iscS* activity have been observed in marine and human gut viromes and contribute to the assembly of iron-sulfur clusters [24, 73]. Iron-sulfur clusters are important in electron transfer as well as comprising the active site of key rumen metalloproteins including hydrogenases [74, 75]. The enzyme *cysH* has been found in viral genomes previously, most notably in temperate phages infecting *Burkholderia* [76, 77]. In the context of *Burkholderia* phages, it has been proposed that *cysH* may serve as a fitness factor during times of sulfur limitation [78]. A similar role for *cysH* during the infection of rumen microbes seems likely. Together, our results show that sulfur AMGs in the rumen may be involved in electron transfer processes, the function of metalloproteins, and sulfate reduction.

## Conclusions

The complex microbial ecosystem of the rumen has a significant impact on the ruminant host’s health and productivity through the breakdown and fermentation of feed into available energy in the form of VFAs. Despite the growing understanding of the functions and importance of viruses in other ecosystems, little is known regarding the roles and dynamics of rumen viruses. Improving our foundational knowledge of the rumen microbial ecosystem will aid in exploiting the roles of members of the rumen ecosystem to increase animal productivity and improve the sustainability of ruminant agriculture. Here, we detailed the distribution and ecological drivers of rumen viruses alongside their bacterial counterparts. Through multivariate approaches, we demonstrated TDN content, a measure of dietary energy, to be the predominant ecological driver of the bacterial and viral response to dietary change. Moreover, we identified OTUs and viral populations that exhibited a linear response along the energy gradient. Together, our results demonstrate niche differentiation of rumen viruses based on dietary conditions.

While viral communities displayed large shifts following dietary perturbation, a fraction of abundant viral populations were found in nearly all viral metagenomes. A core subset of viruses has implications for understanding the general principles of microbial establishment in the rumen and the ubiquitous nature of certain members of the rumen microbiome [3]. More viral metagenomes from an expanded number of animals and ruminant species are needed to identify the composition of the core rumen virome and to better identify putative viral-host relationships among these core microbial populations.

Lastly, we explored the role of viruses in influencing host microbial metabolism through virally encoded AMGs in low and high TDN diets. AMGs in the rumen appear to be reprogramming hosts in a similar fashion to what is described in marine systems. We observed AMGs that redirect carbon flux into the PPP and one-carbon pools through folate metabolism for increased dNTP synthesis and reducing power needed to bolster viral replication. Virally encoded glycosidic hydrolases were also described and are thought to augment energy production during viral infection. The 75 AMGs identified through conservative approaches likely represent the most abundant genes, and further rumen viral studies, especially those that take advantage of long-read technology [79], will elicit more AMGs. It is worth considering that viruses are not the only mobile genetic elements capable of altering host metabolism. Rumen plasmids have been shown to cross phyla and be enriched for niche-specific functions that may provide advantages to their microbial hosts [80]. A more complete characterization of the rumen viromes metabolic potential would be captured through sequencing both extracellular viruses and induced prophages [27]. In summary, this preliminary study addresses fundamental ecological questions of rumen viral ecology, including the redundancy and responsiveness of viruses during various dietary perturbations that provide a necessary foundation for future hypotheses.

## Methods

### Feeding and sample collection

The University of Nebraska-Lincoln Institutional Animal Care and Use Committee approved animal care and management procedures. Servi-Tech Laboratories (Hastings, NE) performed chemical analyses of the diets. Five ruminally fistulated steers (average body weight = 567 kg, age =~ 18 months) were used in a 5 × 4 row-column design to evaluate the role of rumen microbial and viral populations in nutrient metabolism. Steers were maintained on a common diet for 21 days prior to the study to decrease animal-to-animal variation. The animals were randomly assigned to four, 21-day periods. Each diet was fed for a period of 21 days. Steers were fed ad libitum every 4 h from day 1 through day 14 using time feeders to measure ad libitum intake. Steers were restricted to 95% ad libitum days 15–21 to minimize leftover feed.

After 21 days of acclimation to a given diet and prior to feeding at 1100 h, a total rumen evacuation was performed. Rumen contents were mixed, and approximately 1 L of cheesecloth-filtered (four layers of cheesecloth) rumen contents were stored at 4 °C until used for viral purification. Viral purifications were performed within 24 h of collection as described below. In addition, 50 ml of total rumen contents (mixture of solid and liquid fractions of the rumen digesta) were snap frozen in liquid nitrogen and stored at − 80 °C until total DNA extraction for analyzing the microbial fraction of the rumen.

Wireless, submersible pH probes (Dascor Inc., Escondido, CA) were placed into the rumen of each steer to monitor ruminal pH. Each ruminal pH probe was weighted to ensure the electrode remained in the ventral sac of the rumen. Before trial initiation, each pH probe was calibrated by submersing the probes in pH 4 and 7 standard solutions. Ruminal pH was recorded every minute for each period. On day 21 of each period, and prior to feeding the next diet, the pH probes were removed from the rumen for approximately 2 h to download pH data and calibrate the probes. Ruminal pH measurements from each period were adjusted using beginning and ending calibration values to ensure accurate pH measurements. VFA concentrations were measured using a GC, as described previously [81]. VFA and pH data presented represent measurements taken at the same time as rumen sample collection for microbial and viral community analysis. Differences between diets in VFA concentrations and pH were tested using a three-way ANOVA considering diet (fixed effects), period (fixed effects), and animal (random effects).

### Total DNA isolation, library preparation, and sequencing on the ion torrent PGM

Total DNA was extracted from rumen samples (all periods, *n* = 20) using the PowerMax Soil DNA Isolation Kit (MO BIO Laboratories, Inc.) according to the manufacturer’s protocols. After visualization of high molecular weight DNA on a 1% agarose gel, 1 μg of resulting DNA was sheared using the Diagenode Bioruptor Standard sonication device (Diagenode, Inc.). The shearing included 7 cycles of 30 s “ON” and 90 s “OFF” to produce fragments with an average length of ~ 400 bp. Sheared DNA was end repaired using the Quick Blunting Kit (New England Biolabs) according to the manufacturer’s protocols. The resulting blunt-ended DNA was ligated with barcoded Ion Torrent specific A adaptors (5′-CCATCTCATCCCTGCGTGTCTCCGACTCAG-*BARCODE*-GAT-3′) and P1 adaptors (5′-CCTCTCTATGGGCAGTCGGTGAT-3′) using the Quick Ligation Kit (New England Biolabs). The adaptor-ligated DNA was purified using 0.7× volume of Agencourt AMPure XP beads (Beckman Coulter, Inc.) and size selected on 2% E-Gel SizeSelect gels (ThermoFisher Scientific) for fragments of ~ 450–500 bp. Barcoded libraries were pooled based on concentration measured by the Agilent 2100 Bioanalyzer (Agilent Technologies). Clonal amplification and enrichment were performed on the Ion One Touch 2 System according to the manufacturer’s protocols (ThermoFisher Scientific). Microbial metagenome libraries were sequenced using 318 chips (v2) according to Ion PGM Sequencing 400 Kit protocol (ThermoFisher Scientific).

### 16S rRNA amplicon sequencing

Total DNA was amplified with universal primers (515F/806R) to sequence the V4 hypervariable region of the 16S rRNA gene in eubacteria using the paired dual index protocol previously described [82]. PCR reactions contained 1.25 units of Terra PCR Direct Polymerase Mix (Takara Bio), 1X Terra Direct PCR Buffer, 0.4 uM forward and reverse primers, and 20–50 ng of nucleic acid template. Cycling conditions were as follows: an initial denaturation of 98 °C for 30 s; 25 cycles of 98 °C for 15 s, 55 °C for 30 s, and 68 °C for 45 s; and a final extension at 68 °C for 5 min. PCR products were visualized on a 1.5% agarose gel and normalized using the SequalPrep Normalization Plate (Invitrogen). Amplicons were subsequently pooled from each sample, and DNA concentration was estimated from the Agilent 2100 Bioanalyzer (Agilent Technologies) prior to sequencing. Bridge amplification and sequencing (500 cycles, MiSeq Reagent Kit v2 (Illumina, Inc.)) were performed as described by the manufacturer.

### Viral enrichment, purification, and DNA isolation

Rumen samples collected from periods 2–4 (*n* = 15) were centrifuged at 4065×*g* for 30 min at 4 °C to pellet large particles. The resulting supernatant was decanted and tangentially filtered through a 0.2 μm and 100 kDa filter, as described previously [83]. Viral enrichments were stored at − 80 °C until DNA extraction. Prior to DNA extraction, viral enrichments were DNase I treated (0.03 U/μl) for 30 min at 37 °C to remove contaminating free DNA. Following DNase I treatment and inactivation (75 °C for 10 min), DNA was extracted from viral enrichments using the QIAamp UltraSens Virus Kit (Qiagen) according to the manufacturer’s protocol.

### Viral metagenome library preparation and sequencing on the ion torrent PGM

Extracted viral DNA was subjected to second-strand synthesis prior to tagmentation to reduce bias against ssDNA viruses during the transposon library preparation. Briefly, extracted viral DNA was incubated with random hexamer primer (1X, ThermoFisher Scientific) at 70 °C for 5 min followed by quick chill on ice for 5 min. The DNA was then subjected to primer extension. The primer extension reaction contained 0.2 U/μl Klenow Fragment (New England Biolabs), 1X Klenow Fragment Reaction Buffer (New England Biolabs), and 66 μM dNTP (ThermoFisher Scientific). The reaction was incubated at 37 °C for 30 min followed by inactivation of the Klenow Fragment with 10 mM EDTA (ThermoFisher Scientific) and heating at 75 °C for 5 min. Resulting DNA was purified with the MinElute PCR Purification Kit (Qiagen), and libraries were constructed using the MuSeek Library Preparation Kit (ThermoFisher Scientific) in combination with the MuSeek Index Set 1 (ThermoFisher Scientific). In an effort to create libraries with a longer length, DNA was incubated with the transposase enzyme for a shorter time of 1:40 (compared to the recommended 5:00 for ~ 250 bp libraries). Amplification of tagmented DNA fragments was carried out for 12 cycles using recommended cycling conditions. Amplified libraries were purified with the MinElute PCR Purification Kit (Qiagen) and size selected on 2% E-Gel SizeSelect gels (ThermoFisher Scientific) for fragments ~ 350–400 bp. Barcoded libraries were pooled based on concentrations measured by the Agilent 2100 Bioanalyzer (Agilent Technologies). Clonal amplification and enrichment were performed on the Ion One Touch 2 System according to the manufacturer’s protocols (ThermoFisher Scientific). The viral metagenomes were sequenced using 318 chips (v2) according to Ion PGM Hi-Q Sequencing Kit protocols (ThermoFisher Scientific).

### Quality control and taxonomic assignment of 16S rRNA amplicons

Initial quality control of 16S rRNA amplicons was carried out according to the mothur “MiSeq SOP” [82, 84]. Paired reads were merged together and trimmed to a fixed length of 250 bp, removing contigs shorter than this threshold. OTUs were identified with VSEARCH [85] using both de novo and reference-based chimera detection. Using QIIME [86], OTU representative sequences were assigned taxonomy with the “mothur” assignment tool trained on the Greengenes Database (ver. 13_8) [87]. Singleton OTUs and those with classification to *Cyanobacteria*, *Archaea*, or contained the term “mitochondria” were filtered out of the dataset. Representative sequences from OTUs were aligned to the V4 region using the SILVA database (release 128) [88] and mothur [84]. Poorly aligned sequences were removed, and the alignment was used to generate a phylogenetic tree [89]. Normalized OTU median abundances for each diet were added to the phylogenetic tree for visualization using the ggtree package in R [90, 91].

### Quality control of microbial and viral metagenomes

Initial quality control and demultiplexing of sequences were performed using the Torrent Suite Software (v4.0.1, parameters: --barcode-mode 1 --barcode-cutoff 0 --min-read-length 100 --trim-min-read-len 100). Initial quality control steps included retaining reads with an exact match to the barcode during demultiplexing and trimming reads from the 3′ end using a 30 bp sliding window with a quality threshold of 16. For microbial metagenomes, Trimmomatic [92] (version 0.33) was used to trim remaining adaptor sequences and retain sequences with a minimum length of 85 bp. Artificial duplicates were removed using cd-hit-454 [93]. Due to biases associated with transposon tagementation, further quality control steps were used for viral metagenomes to ensure removal of excess sequence duplications, high coverage k-mers, and transposon contamination. Trimmomatic [92] (version 0.33) was used to trim remaining adaptor and transposon sequences, crop sequences at 175 bp (sample from animal 259 on diet 55CS during period two was trimmed to 100 bp due to smaller library size), and retain sequences with a minimum length of 75 bp. Artificial duplicates were removed with cd-hit-454. PRINSEQ [94] was used to identify and remove sequences matching at least one of the following criteria: exact match, exact reverse complement match, DUST score above 7, or reads containing a 7-mer of the flanking transposon sequence from the MuSeek Library Preparation Kit (TGAACTG, GAACTGA, AACTGAC, ACTGACG, CTGACGC, TGACGCA, GACGCAC, ACGCACG, CGCACGA, or GCACGAA). Lastly, a combination of PRINSEQ [94] and QIIME (version 1.9.1) [86] was used to identify and remove prefix duplicates of length 75 bp.

### Deep viral metagenome library preparation and sequencing on the Illumina MiSeq System

Based on dietary TDN content, three viral concentrates from the 55CS and 27CDS diets were selected for deeper sequencing on the Illumina MiSeq System (Illumina, Inc.). Extracted viral DNA was subjected to second-strand synthesis prior to tagmentation to reduce bias against ssDNA viruses during the transposon library preparation, as described above. Resulting DNA was purified using the MinElute PCR Purification Kit (Qiagen), and libraries were constructed using the Nextera XT DNA Library Prep Kit (Illumina, Inc.) according to the manufacturer’s protocols. PCR cleanup and size selection for DNA fragments were performed simultaneously on the Pippin Prep System using 1.5% gel cassettes (Sage Science, Inc.). Purified DNA was concentrated using the Savant DNA 120 SpeedVac (Savant Instruments). Dual-indexed libraries were quantified using the NEBNext Library Quant Kit for Illumina (New England Biolabs) and pooled to a final concentration of 4 nM. Bridge amplification and sequencing (600 cycles, MiSeq Reagent Kit v3 (Illumina, Inc.)) were performed as described by the manufacturer.

### Quality control of deep viral metagenomes

Sequences generated from MiSeq runs were demultiplexed within BaseSapce and processed independently. Briefly, remaining adapter contamination, transposon-associated sequences, the last 25 bp of the forward read, and the last 100 bp of the reverse read were removed with cutadapt [95] (version 1.8.1). The “fastq_filter” command in USEARCH [96] (v8.0.1623) was used to remove sequences containing any ambiguous bases, an expected maximum error rate greater than 0.01, and sequences shorter than 80 bp. A combination of PRINSEQ [94] and QIIME [86] was used to identify and remove prefix duplicates of length 80 bp.

### Estimates of cellular contamination in viral metagenomes

Cellular contamination of viral and deep viral metagenomes was estimated from 16S and 23S rRNA gene sequences detected by the standalone meta_rna software [97].

### Metagenome assembly and ORF prediction

All reads within a dataset (microbial, viral, and deep viral) were assembled independently with SPAdes [98] using unpaired reads as input (k-mer lengths of 21, 33, 55, 77, 99, and 127 were utilized along with the single cell setting (--sc) due to highly non-uniform coverage. ORFs were predicted from contigs longer than 300 bp with Prodigal (metagenomic mode) [99]. ORFs shorter than 60 amino acids were removed and not considered in further analyses.

### Defining viral populations

Reads from the viral and deep viral metagenomes were cross-assembled independently. Contigs longer than 1 kbp were filtered with VirFinder [36] and VirSorter (virome decontamination mode) [37] and used as input for genome binning with Metabat [100] to identify putative viral populations (version 0.32.5; --superspecific --minProb=80 --minBinned=40 --minCV=1 --minContig=1500 --minContigByCorr=500 --minClsSize=10000 -B 20 --pB 20). Binning parameters were chosen that minimized the number of bins with duplicate copies of the *terL* gene. Bins containing multiple *terL* genes were removed, but contigs from these bins longer than 10 kb were designated as their own population. Lastly, viral bins were removed if they contained conserved single copy bacterial genes (identified with CheckM [101]). Abundances of viral populations were summed by mapping sample reads to contigs and normalized based on length of the bin and total number of reads in a given sample. Reads were mapped to contigs using Bowtie 2 [104] (version 2.2.5, default parameters). To assign taxonomy to viral populations, contigs were searched for similarity to the RefSeq viral database (BLASTN, E-value threshold of 10^−5^, bit-score threshold of 50). If multiple contigs from the same viral population had homology to a known virus and were in disagreement, then the taxonomy of the longest contig was assigned to the population. Abundances of viral families were summed from viral populations based on read mapping to contigs and normalized based on the total length of bins assigned to the family and sample read depth (log_2_ counts per kilobase per million [102]). Family abundances were visualized as a heatmap constructed with the Superheat package in R [91, 103]. Rows and columns were clustered by K-means clustering.

### Protein clustering

Inputs for viral PCs were the same contigs used to identify viral populations—those contigs greater than 1 kbp and identified by VirFinder [36] or VirSorter [37] as being of viral origin. ORFs predicted from these viral contigs and microbial ORFs were self-clustered (cd-hit -c 0.6 -aS 0.8 -g 1 -n 4 -d 0 -M 30000; 60% percent identity and 80% coverage). Abundances of PCs were calculated by mapping sample reads to ORFs using Bowtie 2 [104] (version 2.2.5, default parameters). PCs with two or more ORFs or singleton PCs present in a minimum number of 3 samples were considered in downstream analyses. Relative abundance was calculated by dividing the raw PC abundance by the total number of reads in a sample.

### Alpha diversity

Alpha diversity and rarefaction curves were calculated in QIIME [86] based on OTU, viral population, and PC abundances across samples. In the viral metagenomes, one sample—Steer 3244, Corn diet—was an outlier due to lower sequencing depth and removed from alpha diversity analyses. Chao1 and Shannon diversity indices were calculated at an even depth by subsampling to an equal number of sequences per sample. Differences in alpha diversity across samples were tested using a three-way ANOVA considering diet (fixed effects), period (fixed effects), and animal (random effects). Post-hoc pairwise comparisons between diets were done through pairwise *t* tests.

### Beta diversity

The relative abundances of viral populations and PCs were used to calculate Bray-Curtis dissimilarity between samples. For OTU-based beta-diversity, the weighted UniFrac distance was calculated from an OTU table subsampled to 21,228 reads (all 16S rRNA beta-diversity measures were carried out with Bray-Curtis dissimilarities as well to support the findings based on UniFrac distances). The Bray-Curtis dissimilarity or weighted UniFrac distance matrix was used as input for a three-way PERMANOVA where animal (random effect), period (fixed effect), and diet (fixed effect) were used as main effects (adonis function of the vegan package [43]). The assumption of multivariate homogeneity of group dispersions for PERMANOVA was tested by running a one-way ANOVA on the outputs of the betadisper function of the vegan package [43]. The betadisper function reduces the original distances to principal coordinates and measures the multivariate dispersion for a group (i.e., dietary treatment) by calculating the average distance of group members to the group spatial median in multivariate space. Intra-steer and intra-diet Bray-Curtis dissimilarities or weighted UniFrac distances were compared (*t* test) to support the relative contributions of dietary- and host-related factors in structuring bacterial and viral populations. Intra-steer dissimilarities were the dissimilarities or distances calculated among samples collected from the same steer. Similarly, intra-diet dissimilarities were the dissimilarities or distances calculated among samples collected from the same diet.

### Ecological drivers of microbial and viral communities

CAP (capscale function in the vegan package [43]) was used to identify the effects of different dietary components, chemical species, population diversity (measured by Chao1 index and Shannon diversity index), functional diversity (measured by Shannon diversity index), pH, and VFA profiles on the structuring of rumen bacterial and viral communities. Bray-Curtis dissimilarities were calculated from the normalized abundance of viral populations and OTUs (Bray-Curtis dissimilarity explained more variation in the data than weighted UniFrac distances). Due to the potential effects of multicollinearity in multivariate analyses, we reduced the number of features by choosing variables of ecological interest, examining the Pearson correlation between variables, backward variable selection (ordistep command in the vegan package [43]), and assessing variance inflation factors. The significance of CAP models was tested using ANOVA permutation tests for CAP (anova.cca function in vegan package [43]). The marginal effects of each term in the CAP model were assessed using the same ANOVA permutation tests for CAP and including the parameter “by = ‘margin’”.

PLSR (pls package [45]) with cross-validation was used to further investigate the role of dietary components in the structuring of bacterial and viral communities through modeling associations between OTUs and viral populations with dietary factors. Univariate regressions (lm function in R [91]) between normalized OTU (log_2_ counts per million as calculated in edgeR [102]) and viral population abundances (log_2_ counts per kilobase per million as calculated in edgeR [102]) and explanatory variables were utilized to support the strength and direction of the associations identified in PLSR. Univariate regression results were visualized as a heatmap (heatmap.2 function in the gplots package [105]).

### Identification of AMGs

The VirSorter pipeline [37] (virome decontamination mode) was used to identify bona fide viral contigs from contigs longer than 1.5 kbp in deep viral metagenome samples. Amino acid sequences of predicted ORFs from all categories of VirSorter predictions were searched against KEGG [106] (Release 69.0) using UBLAST [93] (v7.0.10; E-value threshold of 10^−5^, bit-score threshold of 50). We used previously published guidelines [23] to define AMGs by removing KOs from the following pathways unless they were additionally involved in carbon metabolism (ko01200), nitrogen metabolism (ko00910), or the PPP (ko00030): homologous recombination (ko03440), mismatch repair (ko03430), aminoacyl-tRNA biosynthesis (ko00970), purine metabolism (ko00230), pyrimidine metabolism (ko00240), DNA replication (ko03030), amino sugar and nucleotide sugar metabolism (ko00520), biosynthesis of amino acids (ko01230), base excision repair (ko03410), cysteine and methionine metabolism (ko00270), and arginine and proline metabolism (ko00330). Functions from these pathways are commonly associated with viral genomes and are not “auxiliary.”

We tested for differences in AMG abundances between 55CS and 27CDS diets in the deep viral metagenomes using DESeq2 [107]. For more accurate normalization, the “sizefactor” parameter in DESeq2 was calculated from the abundances of deep viral metagenome ORFs rather than from AMG abundances. Normalized AMG abundances (log_2_ counts per million as calculated in edgeR [102]) were visualized as a heatmap constructed with the Superheat package in R [91, 103]. Rows and columns were clustered by K-means clustering.

## Additional files

Additional file 1: VFA concentrations and pH measurements at the time of rumen sampling. VFA concentrations (mmol) were measured for acetate, propionate, isobutyrate, butyrate, isovalerate, and valerate. In addition to VFA concentrations, pH measurements were collected at the time of rumen sampling. (CSV 1 kb)

Additional file 2: Amplicon and metagenomic sequencing effort. Number of bases, reads, and cellular contamination after quality control in bacterial 16S rRNA amplicon data and microbial, viral, and deep viral metagenomes. (CSV 1 kb)

Additional file 3: Rarefaction curves of species richness (Chao1 index) in bacterial (A) and viral (B) communities display a plateau with increased sequencing depth. These findings suggest that bacterial OTUs and viral populations were well sampled for assessment of dominant populations across rumen samples. (TIFF 552 kb)

Additional file 4: Alpha diversity comparisons of microbial and viral PCs. No differences in Shannon’s diversity index were found for microbial PCs (A) (*P* > 0.05, three-way ANOVA considering the effects of diet, period, and steer). In contrast, the diversity of viral PCs did alter by diet (*P* < 0.05), but not by host animal or period (*P* > 0.05, three-way ANOVA considering the effects of diet, period, and steer). Letters denote differences in richness and diversity observed when comparing the alpha diversity metrics between pairwise combinations of diets (*P* < 0.05, post-hoc pairwise *t* tests). 27CDS—27% condensed distillers solubles; 40MDGS—40% modified distillers grains plus solubles; 55CS—55% corn silage. (TIFF 172 kb)

Additional file 5: NMDS ordination displaying the influence of diet and host animal on structuring of rumen microbial and viral PCs. Unconstrained ordination analysis was used to visualize beta-diversity based on Bray-Curtis dissimilarity calculated from the distribution of PCs across samples in microbial (A) and viral metagenomes (B). Microbial PCs were found to vary significantly by diet, but not host animal or period (PERMANOVA; diet: *P* = 0.001, *R* ^2^ = 0.259; period: *P* = 0.222, *R* ^2^ = 0.146; steer: *P* = 0.081, *R* ^2^ = 0.222). Diet and host animal in which the sample was collected from were significant influencers on viral PC distribution (diet: *P* = 0.001, *R* ^2^ = 0.270; period: *P* = 0.083, *R* ^2^ = 0.143; steer: *P* = 0.047, *R* ^2^ = 0.288). 27CDS—27% condensed distillers solubles; 40MDGS—40% modified distillers grains plus solubles; 55CS—55% corn silage. 222, 259, 346, 3244, and 3257 represent animal identifiers. (TIFF 510 kb)

Additional file 6: Standard correlation coefficients showing associations between bacterial OTUs (A) and viral populations (B) with independent variables. A PLSR model revealed 172 OTUs and 267 viral populations to have a relationship with the ecological drivers of these communities. To evaluate the strength and direction of associations identified by the PLSR model, OTUs and viral populations were tested for correlation with TDN, protein, zinc, and bacterial diversity via univariate linear regressions. (TIFF 2457 kb)

Additional file 7: AMGs detected in deep viral metagenomes. K number and enzyme names of AMGs identified are provided. (CSV 4 kb)

## Abbreviations

AMG: Auxiliary metabolic gene
CAP: Constrained analysis of principal coordinates
cysH: Phosphoadenosine phosphosulfate reductase
dNTP: Deoxyribonucleotide
F6P: Fructose-6-phosphate
GAP: Glyceraldehyde-3-phosphate
glgA: Glycogen synthase
iscS: Cysteine desulfurase
KEGG: Kyoto Encyclopedia of Genes and Genomes
ORF: Open reading frame
OTU: Operational taxonomic unit
PC: Protein cluster
pfkA: Phosphofructokinase
PLSR: Partial least squares regression
PPP: Pentose phosphate pathway
TDN: Total digestible nutrients
THF: Tetrahydrofolate
treZ: Maltooligosyltrehalose trehalohydrolase
VFA: Volatile fatty acid
