# Supplementary figures and images for: Dietary energy drives the dynamic response of bovine rumen viral communities
Source: Microbiome. 2017 Nov 28;5:155. doi: 10.1186/s40168-017-0374-3 (PMC5704599; doi:10.1186/s40168-017-0374-3)

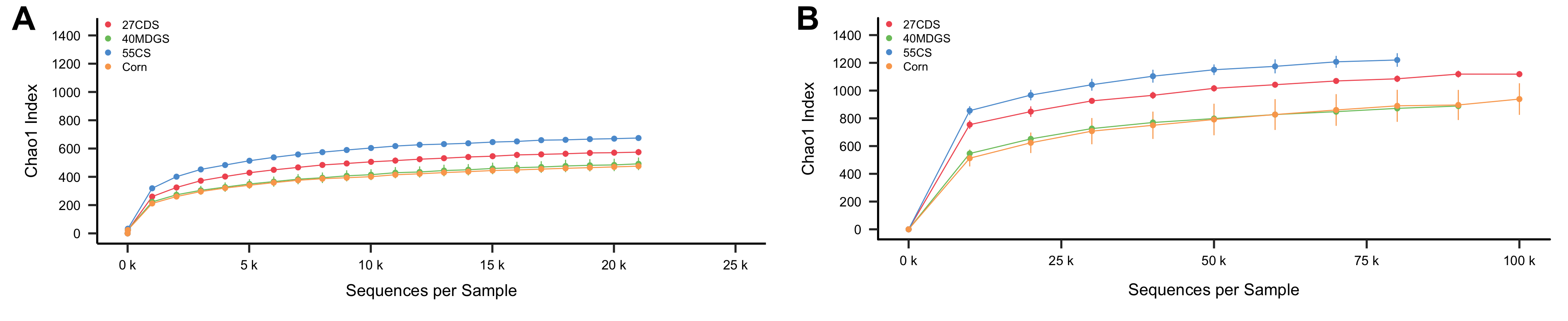

Supplement: Supplementary file 3 — Rarefaction curves of species richness (Chao1 index) in bacterial (A) and viral (B) communities display a plateau with increased sequencing depth. These findings suggest that bacterial OTUs and viral populations were well sampled for assessment of dominant populations across rumen samples. (TIFF 552 kb) [file 40168_2017_374_MOESM3_ESM.tiff]

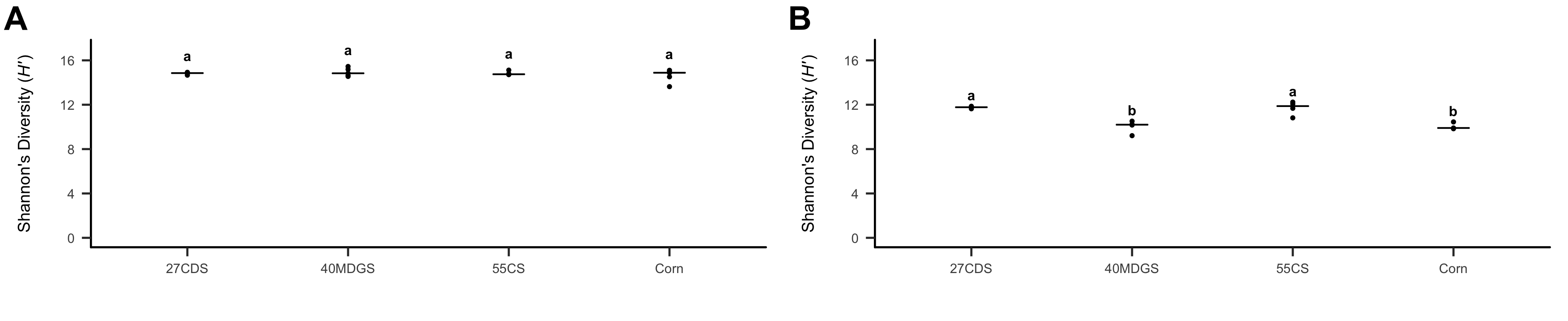

Supplement: Supplementary file 4 — Alpha diversity comparisons of microbial and viral PCs. No differences in Shannon’s diversity index were found for microbial PCs (A) (P > 0.05, three-way ANOVA considering the effects of diet, period, and steer). In contrast, the diversity of viral PCs did alter by diet (P < 0.05), but not by host animal or period (P > 0.05, three-way ANOVA considering the effects of diet, period, and steer). Letters denote differences in richness and diversity observed when comparing the alpha diversity metrics between pairwise combinations of diets (P < 0.05, post-hoc pairwise t tests). 27CDS—27% condensed distillers solubles; 40MDGS—40% modified distillers grains plus solubles; 55CS—55% corn silage. (TIFF 172 kb) [file 40168_2017_374_MOESM4_ESM.tiff]

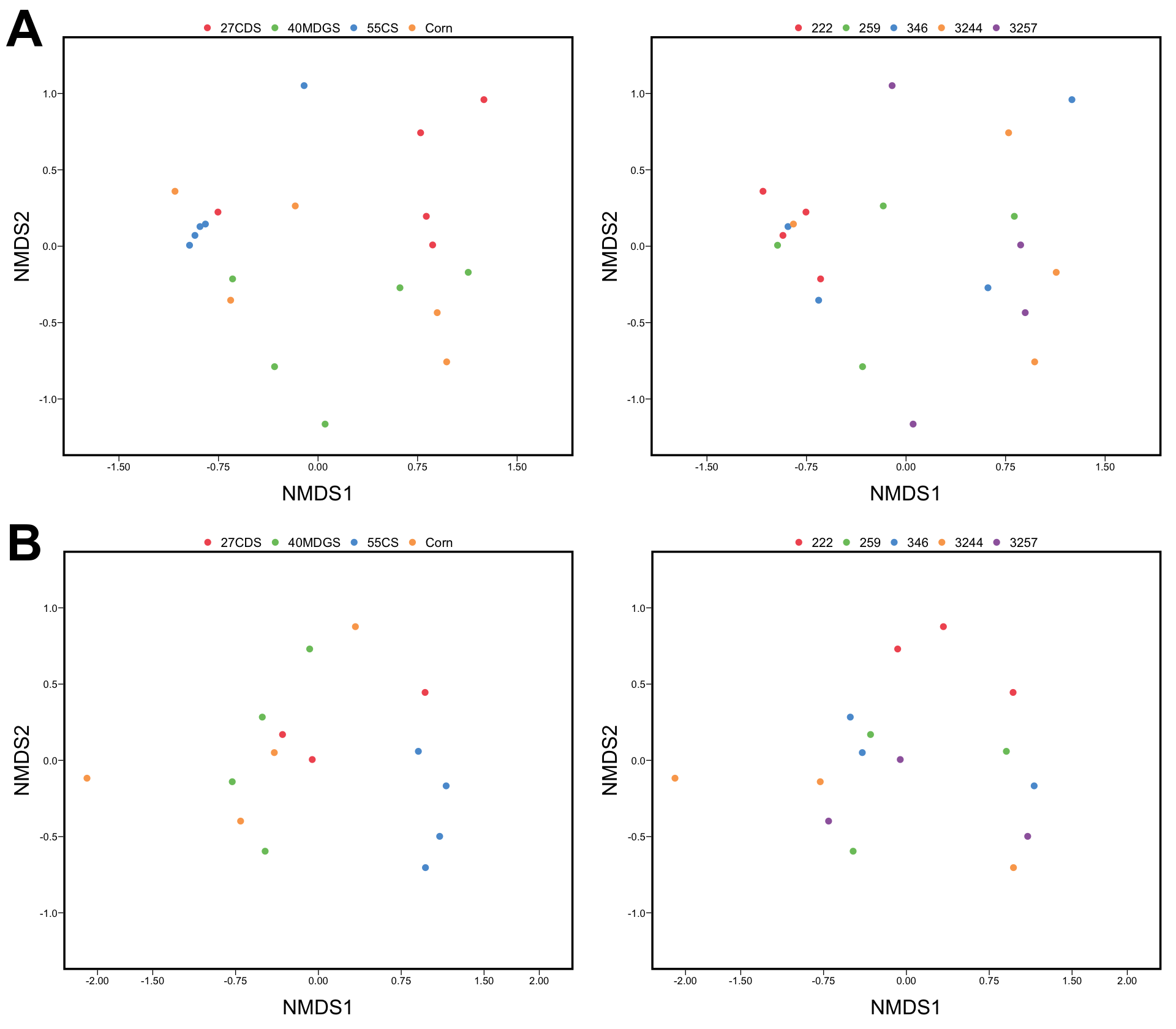

Supplement: Supplementary file 5 — NMDS ordination displaying the influence of diet and host animal on structuring of rumen microbial and viral PCs. Unconstrained ordination analysis was used to visualize beta-diversity based on Bray-Curtis dissimilarity calculated from the distribution of PCs across samples in microbial (A) and viral metagenomes (B). Microbial PCs were found to vary significantly by diet, but not host animal or period (PERMANOVA; diet: P = 0.001, R 2 = 0.259; period: P = 0.222, R 2 = 0.146; steer: P = 0.081, R 2 = 0.222). Diet and host animal in which the sample was collected from were significant influencers on viral PC distribution (diet: P = 0.001, R 2 = 0.270; period: P = 0.083, R 2 = 0.143; steer: P = 0.047, R 2 = 0.288). 27CDS—27% condensed distillers solubles; 40MDGS—40% modified distillers grains plus solubles; 55CS—55% corn silage. 222, 259, 346, 3244, and 3257 represent animal identifiers. (TIFF 510 kb) [file 40168_2017_374_MOESM5_ESM.tiff]

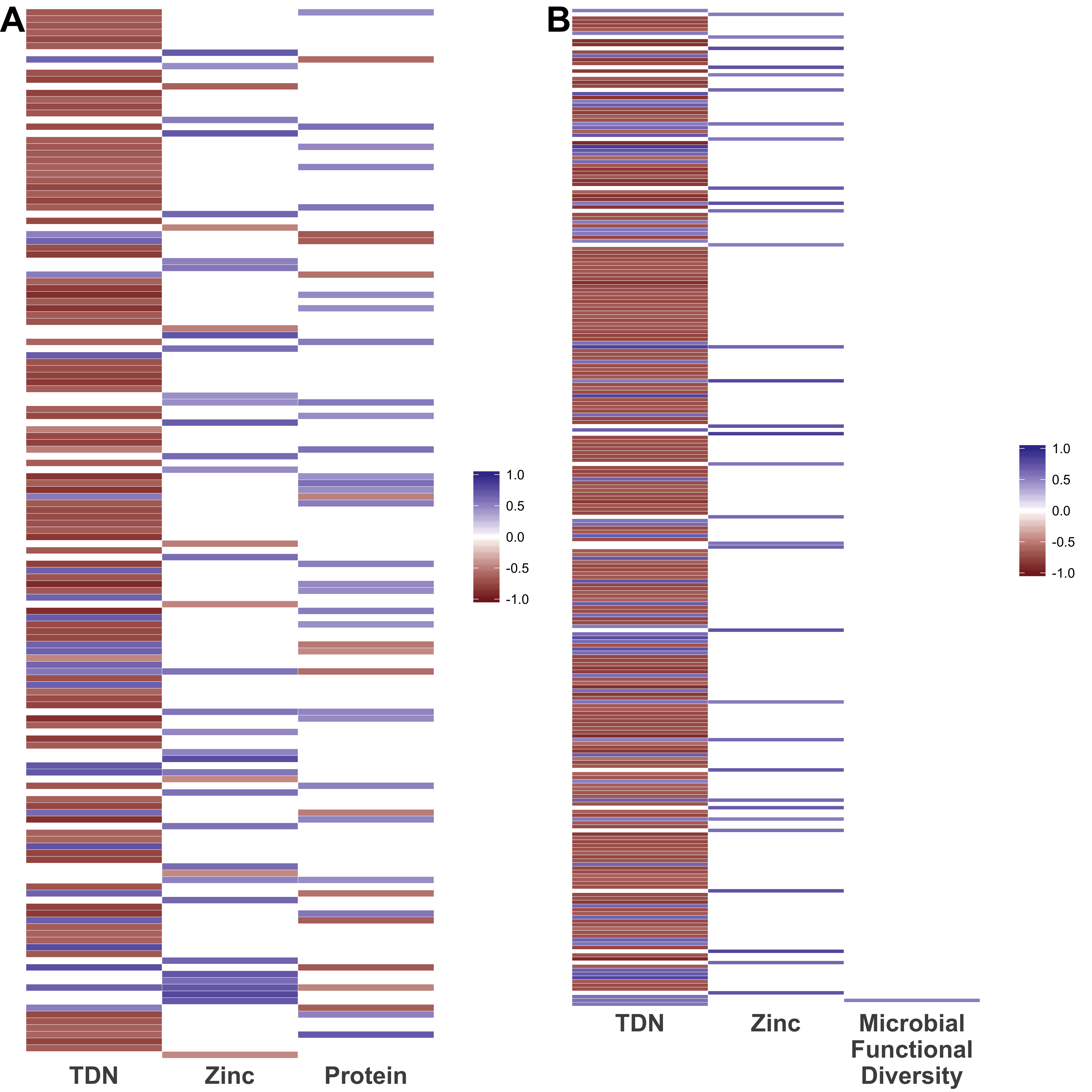

Supplement: Supplementary file 6 — Standard correlation coefficients showing associations between bacterial OTUs (A) and viral populations (B) with independent variables. A PLSR model revealed 172 OTUs and 267 viral populations to have a relationship with the ecological drivers of these communities. To evaluate the strength and direction of associations identified by the PLSR model, OTUs and viral populations were tested for correlation with TDN, protein, zinc, and bacterial diversity via univariate linear regressions. (TIFF 2457 kb) [file 40168_2017_374_MOESM6_ESM.tiff]
